# Sex Hormones and Iron-Related Biomarkers Associate with EMT Features and Tumor Stage in Colorectal Cancer: A Serum- and Tissue-Based Analysis

**DOI:** 10.3390/ijms26115163

**Published:** 2025-05-28

**Authors:** Rosanna Squitti, Anastasia De Luca, Altea Severino, Gianluca Rizzo, Federica Marzi, Luca Emanuele Amodio, Gabriella Vicano, Antonio Focaccio, Vincenzo Tondolo, Mauro Rongioletti

**Affiliations:** 1 Department of Laboratory Science, Research and Development Division, Ospedale Isola Tiberina—Gemelli Isola, 00186 Rome, Italy; altea.severino@fbf-isola.it (A.S.); maurociroantonio.rongioletti@fbf-isola.it (M.R.); 2 Department of Theoretical and Applied Sciences, eCampus University, Viale Massenzio Masia 26, Novedrate, 22100 Como, Italy; 3 Department of Biology, University of Rome “Tor Vergata”, Via della Ricerca Scientifica 1, 00133 Roma, Italy or anastasia.deluca@ptvonline.it (A.D.L.);; 4 Unit of Laboratory Medicine, University Hospital Tor Vergata, Via Montpellier 1, 00133 Rome, Italy; 5 UOC Chirurgia Digestiva e del Colon-Retto, Ospedale Isola Tiberina—Gemelli Isola, 00186 Rome, Italy; gianluca.rizzo@fbf-isola.it (G.R.); federicamarzi9@gmail.com (F.M.); lucaemanueleamodio@virgilio.it (L.E.A.); vincenzo.tondolo@fbf-isola.it (V.T.); 6 Digestive Surgery Unit, Department of Translational Medicine, Catholic University of the Sacred Heart, 00168 Rome, Italy; 7 PhD Program in Cellular and Molecular Biology, Department of Biology, University of Rome “Tor Vergata”, Via della Ricerca Scientifica 1, 00133 Rome, Italy

**Keywords:** colorectal cancer, iron, epithelial to mesenchymal transition (EMT), sex hormones, cancer progression

## Abstract

Sex steroid hormones and systemic iron metabolism are emerging as modulators of colorectal cancer (CRC) development and progression. However, information linking systemic factors to tumor characteristics and epithelial–mesenchymal transition (EMT) is limited, particularly in a sex-specific context. We measured serum levels of sex hormones [testosterone, estradiol, progesterone, Luteinizing Hormone (LH), Follicle-Stimulating Hormone (FSH), Carcinoembryonic antigen (CEA)] and iron-related biomarkers (iron, transferrin, ferritin, % transferrin saturation, ceruloplasmin, and the ceruloplasmin/transferrin ratio) in 82 CRC patients and 31 healthy controls. EMT-related proteins [mediator of ErbB2-driven cell motility 1 (MEMO1), E-cadherin, fibronectin, vimentin, and vinculin] were quantified by Western blotting in tumor and adjacent normal mucosa. Non-parametric tests and Spearman correlations were applied, stratified by sex and corrected for age and anemia where appropriate. Progesterone levels were significantly lower in male CRC patients (median 0.17 ng/mL vs. 0.20 ng/mL, *p* = 0.04) and higher in female patients (0.17 ng/mL vs. 0.10 ng/mL, *p* = 0.0077) compared with controls. The iron-related biomarkers indicated a pattern of iron deficiency, including in non-anemic patients, with reduced % transferrin saturation (*p* < 0.01) and an elevated ceruloplasmin/transferrin ratio (*p* = 0.02). Correlations were found between iron status, tumor stage, and hormonal levels. Progesterone correlated with EMT protein expression in healthy mucosa (e.g., fibronectin in females: ρ = 0.567, *p* = 0.014; vimentin in males: ρ = −0.446, *p* = 0.007), but not in tumor tissue. In the healthy mucosa of male patients, ceruloplasmin/transferrin correlated with MEMO1 (ρ = 0.419, *p* = 0.04), vinculin (ρ = 0.299, *p* = 0.041), and vimentin (ρ = 0.394, *p* = 0.07); transferrin levels inversely correlated with MEMO1 expression (ρ = −0.392, *p* = 0.032), and vimentin showed a positive correlation with serum iron (ρ = 0.350, *p* = 0.043). Furthermore, fibronectin expression inversely correlated with iron in the sole tumor tissue of female patients (ρ = −0.366, *p* = 0.040). These findings support the role of sex hormones and iron metabolism in CRC biology, suggesting that EMT might be accompanied by altered iron uptake and redox remodeling, which can enhance cellular motility and the metastatic potential.

## 1. Introduction

Colorectal cancer (CRC) remains a leading cause of cancer-related morbidity and mortality worldwide, with significant heterogeneity in its pathogenesis, progression, and response to treatment [1]. While most CRC cases arise sporadically, increasing evidence supports a strong influence of systemic factors—including endocrine and metabolic alterations—on tumor behavior and the disease trajectory [2].

Sex differences in CRC incidence and prognosis have long been recognized [3], yet the underlying biological mechanisms remain only partially understood. Steroid hormones such as testosterone, estradiol, and progesterone have been implicated in modulating immune responses, inflammation, and epithelial homeostasis in the gut [4]. Recent experimental studies have also suggested a role for progesterone in regulating the epithelial–mesenchymal transition (EMT), a process central to cancer cell invasiveness and metastasis [5]. However, clinical data on the relationship between systemic hormone levels and EMT-related processes in CRC are still scarce.

In parallel, alterations in iron metabolism have emerged as a hallmark of cancer-related systemic dysfunction. Iron is essential for cell proliferation and DNA synthesis, yet its accumulation or mismanagement can contribute to oxidative stress, immune escape, and ferroptosis resistance [6,7,8]. Clinical studies have shown that patients with CRC often exhibit a functional iron deficiency—characterized by low serum iron and % transferrin saturation despite preserved or elevated ferritin levels—but the implications of these changes in tumor biology remain largely unexplored [9,10,11].

Given these considerations, we hypothesized that systemic alterations in sex steroid hormones and iron-handling proteins may reflect or influence tumor behavior in CRC, possibly through mechanisms involving EMT regulation. To investigate this hypothesis, we analyzed serum levels of sex hormones, iron-related biomarkers, and Carcinoembryonic antigen (CEA) in CRC patients and matched healthy controls. We further assessed the expression of EMT-related proteins in tumor tissues and adjacent healthy mucosa, and explored the associations between circulating factors and tumor clinicopathological features in a sex-stratified approach.

## 2. Results

A total of 113 participants were included in the study, comprising 82 patients with CRC and 31 healthy controls. The CRC group had a significantly higher mean age compared with the controls (69.34 ± 10.8 vs. 60.5 ± 8.8 years, *p* < 0.001, unpaired *t*-test; Table 1). The proportion of female participants was also lower in the CRC group (22%) compared with the controls (34%), although this difference was not statistically significant (*p* = 0.138; chi-square test).

All statistical models included age as a covariate for the male participants, as CRC patients were significantly older than their male controls (69.8 ± 7.09 vs. 53.7 ± 10.5, *p* < 0.01). For female participants, no age correction was applied, since no significant age difference was observed between CRC patients and their controls (69.6 ± 10.8 vs. 66.8 ± 4.4, *p* = 0.326). Since anemia is a common feature of colorectal cancer [12,13,14], hemoglobin levels were assessed and reported in Table 1. To minimize the potential confounding effect of anemia, selected correlation analyses were also performed in the subgroup of non-anemic patients. Among the CRC patients, 51 had anemia (hemoglobin < 12 g/L in females and hemoglobin < 13 g/L in males). Given that 62% of the patients in our cohort presented with anemia, we analyzed the results by both including and excluding anemic individuals. This approach aimed to evaluate the potential impact of anemia on the correlations between iron-related biomarkers and tumor clinical indices.

### 2.1. Comparison of Hormonal and Serum Markers Between CRC Patients and Healthy Controls

We compared serum levels of sex hormones and CEA between CRC patients and healthy controls, stratified by sex. In male participants, values were corrected for age using linear regression residuals, while in females, direct group comparisons were performed using the Mann–Whitney U test.

In females, CRC patients had higher levels of estradiol (*p* = 0.005) and progesterone (*p* = 0.0077) compared with the controls. No differences were observed for Luteinizing Hormone (LH), testosterone, or CEA (Table 2A).

In males, the CRC patients showed significantly lower levels of progesterone (*p* = 0.040). No significant differences were found for Follicle-Stimulating Hormone (FSH), LH, estradiol, testosterone, or CEA (Table 2A).

### 2.2. Comparison of Iron-Related Markers Between CRC Patients and Healthy Controls

Among the female subjects, CRC patients exhibited significantly lower serum levels of iron (35.00 vs. 91.5 µg/dL, *p* < 0.0001), transferrin (216.45 vs. 268.00 mg/dL, *p* < 0.001), and % transferrin saturation (34.89% vs. 12.32%, *p* < 0.001) compared with healthy controls. In contrast, ceruloplasmin levels were significantly higher in the CRC group (31.8 vs. 27 mg/dL, *p* = 0.03). No significant difference was observed for ferritin (Table 2B).

In the male subjects, serum levels of iron were significantly lower in CRC patients compared with the controls (44.00 vs. 101.00 µg/dL, *p* = 0.003). Similarly, transferrin (226.90 vs. 255.90 mg/dL, *p* = 0.0484) and the % transferrin saturation (17.09% vs. 28.54%, *p* = 0.0091) were significantly reduced in the CRC group. In contrast, ceruloplasmin (28.71 vs. 24.65 mg/mL, *p* = 0.02) and the ceruloplasmin/transferrin ratio (1.3 vs. 1.03, *p* = 0.04) were significantly increased; no significant differences were observed for ferritin in both sexes (Table 2B).

To evaluate the potential impact of anemia on the comparison of iron-related biomarkers, we repeated the comparison between CRC patients and healthy controls but considering only non-anemic individuals (defined as Hb ≥ 12 g/dL in women and ≥13 g/dL in men). Among the females, CRC patients showed significantly lower levels of serum iron (*p* = 0.0016), transferrin (*p* = 0.024), % transferrin saturation (*p* = 0.0019), and ceruloplasmin/transferrin ratio (*p* = 0.024), while no difference was observed in ceruloplasmin levels (*p* = 0.1059). Among the males, after adjusting for age, CRC patients had a trend of lower iron (*p* = 0.0538) and higher levels of ceruloplasmin (*p* = 0.022), whereas differences in serum ferritin, transferrin, and % transferrin saturation were not statistically significant (all *p* > 0.10).

### 2.3. Correlations Between Tumor Clinical Indices and Serum Levels of Sex Hormones

**Figure 1:**
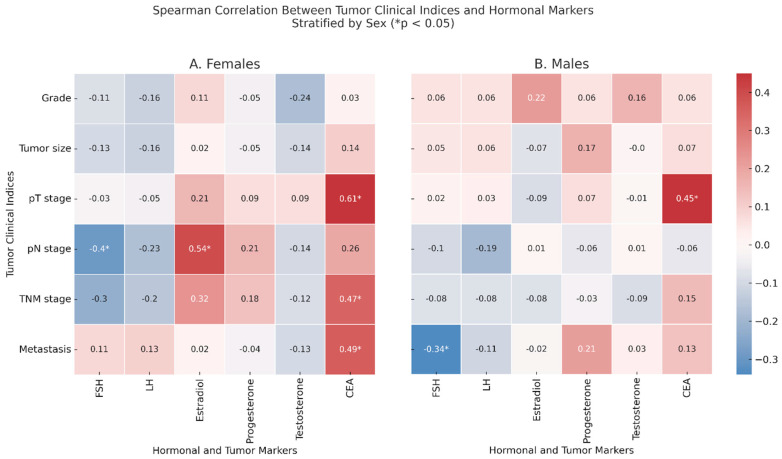
Spearman correlation between tumor clinical indices and serum hormonal markers in colorectal cancer patients, stratified by sex. Panel (**A**) shows the correlation matrix for the female patients. In Panel (**B**) (male patients), age adjustment was applied: residuals from linear regression models including age as a covariate were used prior to Spearman correlation analysis. Tumor clinical indices are reported on the *y*-axis and include the tumor grade, tumor size, pathological T and N stages, overall TNM stage, and presence of distant metastases. Hormonal markers on the *x*-axis include FSH, LH, estradiol, progesterone, testosterone, and carcinoembryonic antigen (CEA). Color intensity reflects the strength and direction of the Spearman correlation coefficient (ρ). Asterisks (* *p* < 0.05) indicate statistically significant correlations.

Spearman correlation analyses were performed to investigate associations between tumor clinical indices and serum levels of sex hormones and CEA in CRC patients, stratified by sex (Figure 1).

In female patients (Figure 1, Panel A), unadjusted analyses revealed that pN was positively correlated with FSH (ρ = −0.4, *p* = 0.030) and with estradiol (ρ = 0.54, *p* = 0.002). Additionally, CEA correlated positively with the pT stage (ρ = 0.61, *p* < 0.001), TNM stage (ρ = 0.47, *p* = 0.008), and with metastasis (ρ = 0.49, *p* = 0.007).

In male patients (Figure 1, Panel B), after correcting for age, FSH was negatively correlated with metastasis (ρ = −0.34, *p* = 0.04), and CEA remained significantly associated with the pT stage (ρ = 0.45, *p* = 0.001). When restricting the analysis to non-anemic CRC patients, several correlations previously observed were no longer statistically significant. In females, the associations between the pN stage and FSH and estradiol levels and between metastasis and CEA lost significance. However, estradiol remained significantly correlated with the tumor grade (ρ = −0.76, *p* = 0.026), and the correlation between the TNM stage and CEA became stronger (ρ = 0.71, *p* = 0.049). In males, the association between FSH and metastasis disappeared, while the correlation between the pT stage and CEA increased (ρ = 0.52, *p* = 0.036).

### 2.4. Correlations Between Tumor Clinical Indices and Iron-Related Markers

**Figure 2:**
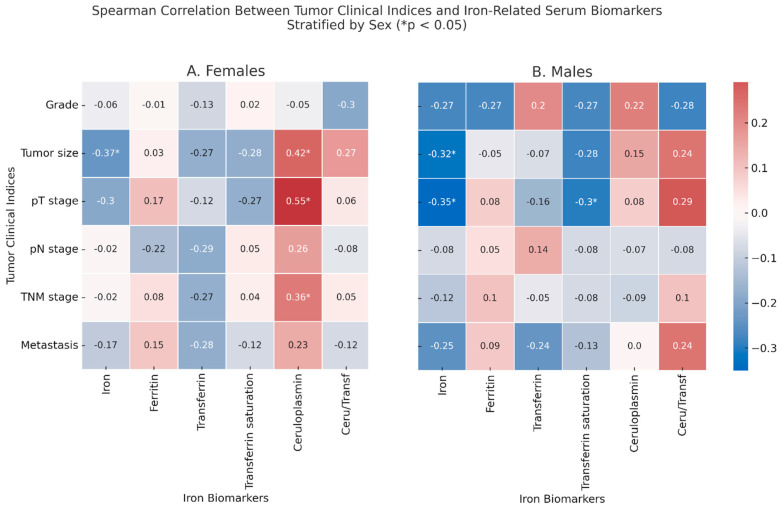
Spearman correlation between tumor clinical indices and iron-related serum biomarkers in colorectal cancer patients, stratified by sex. Panel (**A**) shows the correlation matrix for the female patients. In Panel (**B**) (male patients), age adjustment was applied: residuals from linear regression models including age as a covariate were used prior to Spearman correlation analysis. Tumor clinical indices are reported on the *y*-axis and include the tumor grade, tumor size, pathological T and N stages, overall TNM stage, and presence of distant metastases. Iron-related serum biomarkers on the *x*-axis include serum iron, ferritin, transferrin, % transferrin saturation, ceruloplasmin, and the ceruloplasmin/transferrin ratio. Color intensity reflects the strength and direction of the Spearman correlation coefficient (ρ). Asterisks (* *p* < 0.05) indicate statistically significant correlations.

Among the females (Figure 2, Panel A), tumor size was inversely correlated with serum iron (ρ = −0.37, *p* = 0.046) and positively correlated with ceruloplasmin (ρ = 0.42, *p* = 0.021), which correlated also with the pT stage (ρ = 0.55, *p* = 0.002) and with the TNM stage (ρ = 0.36, *p* = 0.049). In males, the tumor size correlated with lower levels of iron (ρ = −0.32, *p* = 0.046), which inversely correlated also with the pT stage (ρ = −0.35, *p* = 0.044), which was itself inversely correlated with % transferrin saturation (ρ = −0.30, *p* = 0.048). In non-anemic CRC patients, several correlations between iron-related serum biomarkers and hormonal markers or CEA were detected, although only a few reached statistical significance: in males, iron correlated with the tumor size (ρ = −0.57, *p* = 0.020), while transferrin correlated with the pT stage (ρ = −0.524, *p* = 0.045).

### 2.5. Correlation Between Iron-Related Markers and Hormonal/Biochemical Biomarkers

**Figure 3:**
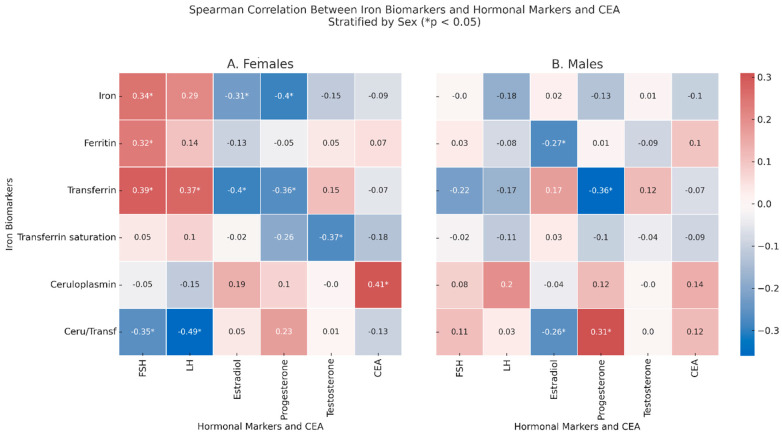
Spearman correlation between serum iron-related biomarkers and hormonal markers, including CEA, in colorectal cancer patients, stratified by sex. Panel (**A**) shows the correlation matrix for the female patients. In Panel (**B**) (male patients), age adjustment was applied: residuals from linear regression models including age as a covariate were used prior to Spearman correlation analysis. Iron biomarkers are reported on the *y*-axis and include serum iron, ferritin, transferrin, the % transferrin saturation, ceruloplasmin, and the ceruloplasmin/transferrin ratio. Hormonal markers and carcinoembryonic antigen (CEA) are shown on the *x*-axis and include FSH, LH, estradiol, progesterone, testosterone, and CEA. Color intensity represents the strength and direction of the Spearman correlation coefficient (ρ). Asterisks (* *p* < 0.05) denote statistically significant correlations.

In the females (Figure 3, Panel A), significant correlations were observed between FSH and iron (ρ = 0.34, *p* = 0.19), ferritin (ρ = 0.32, *p* = 0.033), and transferrin (ρ = 0.39, *p* = 0.07), while there was an inverse correlation with ceruloplasmin/transferrin ratio (ρ = −0.35, *p* = 0.043), which inversely correlated with LH (ρ = −0.49, *p* = 0.003), which also correlated with transferrin (ρ = 0.37, *p* = 0.014). Estradiol correlated with lower levels of transferrin (ρ = −0.4, *p* = 0.005) and iron (ρ = −0.31, *p* = 0.035); testosterone correlated with lower values of % transferrin saturation (ρ = −0.37, *p* = 0.032). Finally, CEA correlated with increased levels of ceruloplasmin (ρ = 0.41, *p* = 0.015). In the males (Figure 3, Panel B), after adjusting for age, ferritin showed a negative correlation with estradiol (ρ = −0.27, *p* = 0.048), and transferrin inversely correlated with progesterone (ρ = −0.36, *p* = 0.045). Moreover, the ceruloplasmin/transferrin ratio correlated with estradiol (ρ = −0.26, *p* = 0.048) and with progesterone (ρ = 0.31, *p* = 0.04).

In non-anemic CRC patients, some of the previously observed correlations between iron-related biomarkers and hormonal markers disappeared, while new significant associations emerged.

In the females, the correlations between % transferrin saturation and testosterone and between the ceruloplasmin/transferrin ratio and FSH were no longer significant. However, new associations were detected: in females, serum iron inversely correlated with progesterone (ρ = −0.55, *p* = 0.012), and transferrin also showed a negative correlation with progesterone (ρ = −0.45, *p* = 0.046); finally, LH was negatively correlated with the ceruloplasmin/transferrin ratio (ρ = −0.738, *p* = 0.037). In the males, the previous associations between the ceruloplasmin/transferrin ratio and estradiol or progesterone were no longer evident. Instead, new significant correlations emerged: in males, ceruloplasmin positively correlated with CEA (ρ = 0.38, *p* = 0.032), and the ceruloplasmin/transferrin ratio was strongly and inversely associated with CEA (ρ = −0.54, *p* = 0.002). LH was negatively correlated with both serum iron (ρ = −0.62, *p* < 0.001) and % transferrin saturation (ρ = −0.50, *p* = 0.005).

### 2.6. Comparison of EMT Marker Expression in Tumor and Matched Healthy Mucosa

**Figure 4:**
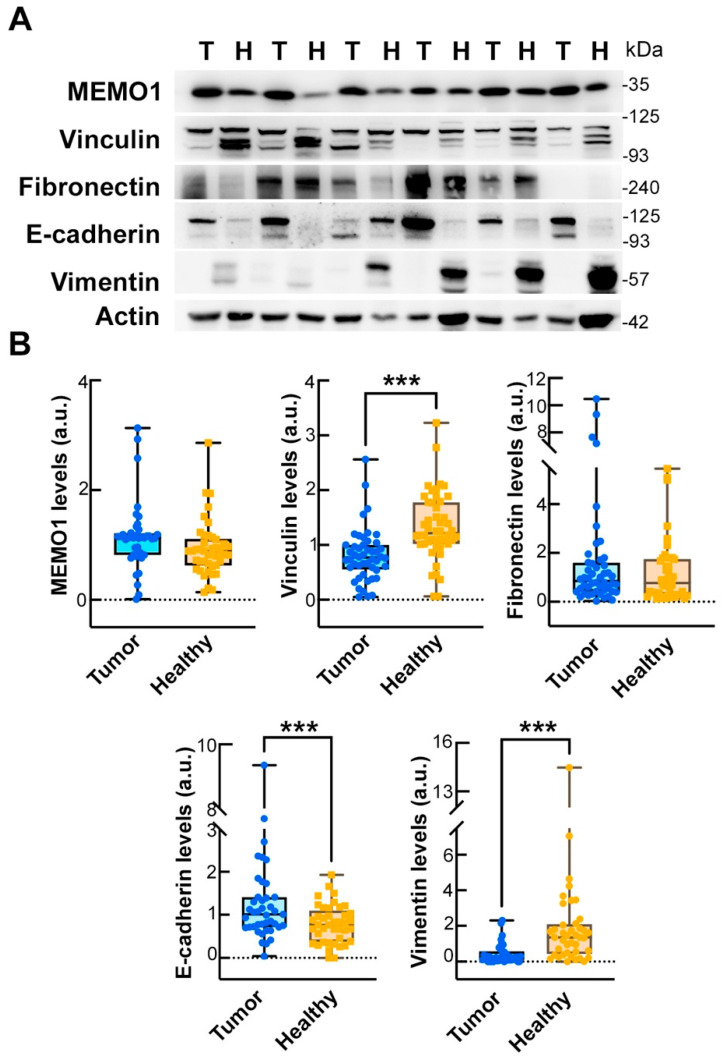
Analysis of the levels of EMT hallmarks in CRC patients. (**A**) Representative Western blot images of the levels of the EMT proteins MEMO1, vinculin, fibronectin, E-cadherin, and vimentin obtained from six patients. Protein levels in the cancerous tissues (T) were compared with those in their matched healthy mucosa (H). β-actin was used as a loading control. (**B**) Densitometric analyses of MEMO1, vinculin, fibronectin, E-cadherin, and vimentin. The data are presented as box and whiskers boxplots showing the single values obtained from each patient. Mann–Whitney test: *** *p* < 0.001.

We analyzed the expression of key EMT biomarkers in paired samples of tumor tissue and adjacent healthy mucosa from CRC patients. Wilcoxon signed-rank tests were used to compare the distributions of protein expression between the two tissue types. A significant decrease in vinculin expression was observed in tumor tissue compared with healthy mucosa (mean ± SD: 0.75 ± 0.51 vs. 1.46 ± 0.89, *p* < 0.001; Table 3 and Figure 4), as well as in vimentin (0.78 ± 0.80 vs. 4.08 ± 4.88, *p* < 0.001; Table 3 and Figure 4). Conversely, E-cadherin levels were significantly higher in the tumor samples (1.44 ± 0.72 vs. 0.81 ± 0.45; *p* < 0.001; Table 3 and Figure 4). No significant differences were found for mediator of ErbB2-driven cell motility 1 (MEMO1) or fibronectin (*p* = 0.729 and 0.259, respectively).

### 2.7. Association Between Sex Hormones and EMT Proteins in Normal Mucosa and Tumor Tissue

We investigated the relationship between circulating progesterone levels and the expression of EMT-related proteins in both the healthy mucosa and tumor tissue of colorectal cancer patients, stratified by sex. In the healthy mucosa, significant correlations were observed in the female patients: serum progesterone was positively correlated with fibronectin expression (ρ = 0.567, *p* = 0.014). In the male patients, serum progesterone showed a negative correlation with vimentin expression (ρ = −0.446, *p* = 0.007). These associations, however, were no longer observed in tumor tissue, where no significant correlations between progesterone levels and EMT protein expression were detected in either sex.

### 2.8. Association Between Iron-Related Biomarkers and EMT Proteins in Normal Mucosa and Tumor Tissue

The pattern of correlations between EMT-related proteins and iron metabolism markers differed between tumor tissue and healthy mucosa and also varied according to sex.

In the healthy mucosa of male patients, the ceruloplasmin/transferrin ratio correlated with MEMO1 (ρ = 0.419, *p* = 0.04), vinculin (ρ = 0.299, *p* = 0.041), and vimentin (ρ = 0.394, *p* = 0.07). Furthermore, transferrin levels inversely correlated with MEMO1 expression (ρ = −0.392, *p* = 0.032), and vimentin showed a positive correlation with serum iron (ρ = 0.350, *p* = 0.043).

However, in tumor tissue, all these associations were lost, except for a preserved inverse correlation between vinculin and transferrin (ρ = −0.355, *p* = 0.040). In healthy female mucosa, no significant correlations were observed between iron-related biomarkers and EMT proteins. In contrast, in tumor tissue from female patients, a significant negative correlation emerged between iron and fibronectin expression (ρ = −0.366, *p* = 0.040).

## 3. Discussion

The most important result of our study is the observation that serum progesterone levels are significantly associated with the expression of EMT-related proteins in non-tumoral colorectal mucosa but not in the tumor tissue itself. Specifically, higher progesterone levels correlated with increased fibronectin in females and decreased vimentin in males. These sex-specific associations were no longer detectable in cancer tissue, suggesting that the link between systemic hormonal signaling and local EMT expression is disrupted during malignant transformation.

The epithelial–mesenchymal transition, as assessed in this study by protein expression, represents a key biological process that contributes to cancer cell plasticity, invasiveness, and metastatic potential. We observed changes in EMT-related proteins that were consistent with a shift toward a less adhesive, more invasive phenotype, as revealed by Western blot analysis. These findings are supported by our previous study [16], in which similar EMT alterations were confirmed at the transcript level by RT-qPCR, thereby reinforcing the biological relevance of the protein-level observations.

Our results support the hypothesis that progesterone may exert a protective role in the early phases of carcinogenesis by modulating EMT-related proteins in the normal mucosa, as proposed in the literature [5,17,18,19,20]. This suggestion is aligned with preclinical data from Zhang et al. (2021) [5], who demonstrated that progesterone suppresses proliferation, migration, and EMT in CRC cells through the activation of GADD45α and inhibition of JNK signaling. Their study provides a mechanistic explanation that complements our clinical findings, suggesting that the GADD45α/JNK pathway may also mediate the hormone–EMT interaction in vivo. However, it is important to acknowledge that progesterone may exert pro-tumorigenic effects in the presence of specific receptors such as PGRMC1 [21].

In our sex-stratified correlation analysis, several serum biomarkers demonstrated significant associations with clinicopathological features of colorectal cancer. In male patients, LH levels were positively correlated with the presence of metastasis, while CEA levels correlated with the TNM stage in both sexes. In females, FSH and estradiol levels were associated with the pN stage, potentially reflecting sex hormones’ modulation of immunological factors. Among iron-related markers, ceruloplasmin and the ceruloplasmin/transferrin ratio were significantly associated with tumor stage (pT), tumor size, metastasis, and tumor grade, particularly in males. Notably, iron deficiency was more pronounced in men, where it also correlated with advanced tumor stage (pT). These findings highlight the functional relevance of systemic biological profiles in CRC behavior and support the hypothesis that alterations in the hormone and iron-related biomarkers are not merely epiphenomena but may contribute to the pathophysiological processes driving tumor aggressiveness. Additionally, our results are consistent with large-scale epidemiological studies indicating a protective role for endogenous sex hormones in CRC. For instance, Lin et al. (2013) [4] found that higher circulating testosterone and Sex Hormone-Binding Globulin (SHBG) levels were associated with lower CRC risk in men, while Harbs et al. (2022) [3] further emphasized sex-based differences in hormonal pathways influencing tumor biology and the response to inflammation. The opposite trends observed in serum progesterone levels between male and female CRC patients suggest a sex-specific dysregulation of steroidogenic pathways during tumorigenesis. While lower progesterone in males may reflect a deficiency in protective hormonal tone, higher levels in females might indicate an ineffective compensatory response or altered metabolism associated with tumor-related inflammation. Together, these findings highlight a disruption of physiological endocrine–epithelial cross-talk in CRC and point toward the hormone–EMT axis as a promising focus for further mechanistic and therapeutic investigations.

Testosterone levels were lower in male CRC patients (median 14.32) compared with healthy controls (median 17.69), with a trend toward significance (*p* = 0.052; Mann–Whitney test). However, this difference was no longer significant after adjusting for age. Despite this, our findings align with previous epidemiological studies reporting an inverse association between circulating testosterone and the CRC risk in men [3,4], supporting the hypothesis that androgens may exert a protective role in colorectal carcinogenesis.

The second key finding of this study is the observation of consistent alterations in serum iron-related biomarkers in patients with colorectal cancer. CRC patients showed reduced % transferrin saturation and an elevated ceruloplasmin/transferrin ratio, indicating a systemic shift in iron distribution and transport. These changes were observed across sexes and were independent of anemia-related markers, suggesting a cancer-specific iron imbalance rather than a reflection of general nutritional status. Lower % transferrin saturation may reflect iron sequestration or restricted availability, potentially contributing to tumor-driven metabolic adaptations. Along these lines, Sawayama et al. (2021) [22] identified low pre-operative transferrin levels as an independent predictor of worse survival in CRC, reinforcing the clinical relevance of systemic iron status as a prognostic factor. Our data similarly suggest that iron-related markers could serve as accessible biomarkers for patient stratification and prognosis in CRC. The ceruloplasmin/transferrin ratio further underscores a disruption in iron-handling pathways in CRC. Our results show that CRC patients had a significant increase in the ceruloplasmin/transferrin ratio. This parameter reflects the oxidative loading of iron (Fe^2+^ to Fe^3+^) onto transferrin by ceruloplasmin, facilitating its systemic transport and cellular uptake. Its elevation may indicate a tumor-driven shift in iron homeostasis, favoring iron redistribution and limiting free-iron toxicity, potentially supporting tumor growth and survival [23,24]. As an integrative marker, the ceruloplasmin/transferrin ratio may serve as a candidate biomarker for future risk stratification or disease monitoring. As a whole, compared with healthy controls, the CRC patients exhibited a consistent profile characterized by reduced % transferrin saturation and increased ceruloplasmin/transferrin ratio, both in males and females. These alterations suggest a functional iron deficiency, despite largely normal ferritin levels—an observation that mirrors the pattern described in recent clinical cohorts [9,10,11,24]. This condition has been attributed to tumor-related chronic inflammation, impaired absorption, and hidden blood loss and is prevalent in CRC patients even before anemia becomes clinically evident [9,10,11,24]. Given that anemia is a common feature of colorectal cancer [12,13,14], we addressed its potential confounding effect by performing correlation analyses restricted to non-anemic patients. This strategy aimed to clarify whether the associations observed were directly linked to biological processes independent of anemia. However, this approach considerably reduced the sample size, particularly in the stratified analyses. Therefore, the results obtained in the non-anemic subgroup should be interpreted with caution, as the lack of statistical significance may reflect either a true absence of anemia-related influence or a loss of statistical power due to the limited sample size. Nevertheless, the results in non-anemic patients confirm that iron-related biomarker alterations in CRC are not solely attributable to anemia and likely reflect disease-specific dysregulation. In our study, we observed significant associations between testosterone, LH, and markers of iron metabolism and tumor stage (pT) in male CRC patients. Lower levels of iron and % transferrin saturation with increased levels of LH in non-anemic patients suggest systemic changes that influence iron handling; specifically, sex hormones may modulate extracellular iron availability in the tumor microenvironment, possibly by affecting hepatic transferrin expression or iron sequestration by tumor cells [25,26].

As a whole, our findings suggest a state of functional iron deficiency, likely driven by tumor-associated inflammation and hepcidin-mediated iron sequestration, rather than overt iron store depletion. Such alterations are consistent with clinical data reporting that iron deficiency—with or without anemia—is present in up to 60% of CRC patients. Interestingly, this state of systemic iron dysregulation may not simply reflect the tumor burden but potentially contribute to cancer progression. As Aksan et al. [24] extensively reviewed, iron deficiency impairs immunosurveillance, weakens anti-tumor immunity, and fosters hypoxia-induced responses such as HIF-1α activation, angiogenesis, and EMT-like behavior. These mechanisms align with our own findings linking iron-related biomarkers to the tumor stage and EMT marker expression, suggesting a role for iron in shaping the tumor microenvironment and influencing malignant transformation. Along the same lines, we observed significant positive correlations in the healthy mucosa from male patients, mainly between the ceruloplasmin/transferrin ratio and EMT markers such as MEMO1, vinculin, and vimentin, that were largely lost in the tumor tissue, except for a new inverse correlation between vinculin and transferrin. In female patients, a significant negative correlation emerged between iron and fibronectin expression. These findings suggest that the relationship between iron metabolism and EMT markers is both tissue- and sex-specific. In healthy male mucosa, positive correlations between the ceruloplasmin/transferrin ratio and EMT markers suggest a coordinated regulation, while their loss in tumor tissue implies a disruption during CRC progression [24,27,28]. In females, the tumor-specific negative correlation between iron and fibronectin, absent in healthy tissue, highlights a sex-specific shift in iron–ECM dynamics during tumorigenesis [24,27,28]. Sex hormones such as testosterone and estradiol regulate systemic and cellular iron metabolism by modulating the expression of key proteins including ferroportin (FPN) and divalent metal transporter 1 (DMT1), thereby influencing iron uptake, storage, and export [29,30,31]. This hormonal regulation may impact the iron availability within the tumor microenvironment, affecting CRC progression.

The role of iron-deficiency anemia (IDA) in colorectal cancer has also been examined in large clinical studies. Almilaji et al. (2021) [9] reported that CRCs presenting via the IDA diagnostic pathway are more frequently right-sided and, paradoxically, more likely to be diagnosed at an early stage, likely due to proactive diagnostic workups prompted by anemia detection. These findings emphasize that IDA may serve as both a clue to early detection and a marker of a specific CRC phenotype, especially in the right colon [9,11].

Overall, our findings provide additional support for the emerging notion that iron deficiency is not a neutral bystander but rather an active player in tumor biology. Whether by altering immune homeostasis, promoting oxidative stress, or modifying EMT programs, iron dysregulation may shape cancer behavior and prognosis, and it deserves further exploration both as a biomarker and potential therapeutic target.

Recent studies have revealed that CRC cells may adapt their iron metabolism not only to support proliferation but also to escape ferroptosis, a regulated cell death mechanism driven by iron-dependent lipid peroxidation [6,7,8,32,33,34,35,36]. Ferroptosis resistance has been associated with increased antioxidative buffering (e.g., GPX4 activity), decreased iron availability, or altered expression of iron-handling proteins. In this context, our finding of reduced % transferrin saturation and elevated ceruloplasmin/transferrin ratios in CRC patients may reflect a tumor-associated shift aimed at limiting ferroptotic vulnerability [6,7,8,32,33]. Moreover, ferroptosis has been shown to interface with EMT signaling, as mesenchymal-like cancer cells often exhibit reduced sensitivity to ferroptosis. The systemic iron phenotype observed in our CRC cohort may thus be functionally linked to the EMT features described in tumor tissue, supporting the idea that iron restriction contributes not only to immune modulation and oxidative balance, but also to tumor cell plasticity and survival [7,8,33]. The epithelial–mesenchymal transition has been associated with altered cellular metabolism, including increased iron uptake and ROS production, supporting enhanced motility and the invasive capacity of cancer cells [37,38]. This metabolic remodeling may partly explain the associations we observed between EMT markers and iron status in CRC tissues.

Another result concerns the CEA trend in sex-stratified comparisons: in our study, CEA levels were significantly higher in CRC patients compared with controls in both sexes, but the difference was more pronounced in males. This observation may reflect a sex-based variation in tumor biology, systemic inflammation, or CEA metabolism. Although CEA remains a widely used biomarker in CRC, our findings highlight potential sex-specific variability in its diagnostic performance, which may warrant further stratified analyses in clinical settings [39].

Taken together with our findings on CEA and hormonal/metabolic markers, these results highlight the value of integrating multiple systemic biomarkers to better stratify patients. A multimarker approach combining CEA, iron-related biomarkers, and sex steroid hormones could enhance early detection, monitor disease progression, and personalize treatment strategies, especially when adjusted for sex-specific differences in physiology and tumor behavior.

This study has several limitations. First, the cross-sectional design does not allow for the inference of causality between systemic biomarkers and tumor progression. Second, the EMT analysis was performed only at the protein level by Western blotting, without transcriptional or spatial resolution. However, in a previous study from our group, EMT protein expression was validated at the transcript level by RT-qPCR, albeit in a smaller subset of patients [16]. Third, SHBG levels were not measured, limiting the interpretation of hormone bioavailability. Fourth, although all healthy controls were aged over 50 years, the CRC group was significantly older among males. To address this, we applied age correction in all relevant analyses, and male sex hormone levels are generally stable after age 50, minimizing the risk of age-related bias. Finally, the sample size, particularly for tissue-based analyses, remains modest and requires validation in larger and prospective cohorts.

## 4. Materials and Methods

### 4.1. Subjects

This study was conducted in accordance with the ethical standards of the Declaration of Helsinki (1964) and its subsequent revisions. Ethical approval was obtained from the Institutional Ethics Committee (CET Lazio, Area 3) of the Fondazione Policlinico Universitario Agostino Gemelli IRCCS—Università Cattolica del Sacro Cuore (protocol no. 6091/2023, approved on 26 October 2023). All participants provided written informed consent for the use of their biological samples and clinical data.

Eighty-two consecutive patients with a diagnosis of colorectal cancer were recruited at the Digestive and Colorectal Surgery Unit of the Isola Tiberina Hospital—Gemelli Isola between October 2023 and February 2024. Inclusion criteria for CRC patients included age > 18 years and a confirmed diagnosis of colorectal adenocarcinoma based on both endoscopic and histopathological evaluation in accordance with the 2023 NCCN Guidelines for Colon Cancer. Upon diagnosis, patients underwent standard staging procedures to assess their tumor extension and surgical eligibility.

The control group consisted of 31 healthy volunteers recruited from among blood donors at the Ematos–Fidas Unit of the same institution between October 2023 and November 2023. Individuals with severe medical conditions (including diabetes mellitus, chronic heart or respiratory failure, hepatic or renal impairment, recent alcohol abuse, or cognitive disorders) were excluded from both groups. In addition, subjects were screened for anemia, defined as hemoglobin levels < 12 g/dL in females and <13 g/dL in males, to assess the potential impact of anemia on the comparison of iron-related biomarkers.

### 4.2. Western Blot Analysis of Tissue Homogenates

Protein extraction from tissue homogenates was followed by quantification using the Lowry-based DC™ Protein Assay Kit (Bio-Rad, Hercules, CA, USA). Aliquots of samples were mixed with 4× Laemmli buffer supplemented with 5% β-mercaptoethanol and denatured at 95 °C for 5 min. Fifty micrograms of total protein per sample were resolved by SDS–PAGE on 8%, 10%, or 12% polyacrylamide gels, depending on the molecular weight of the target proteins, and subsequently transferred onto PVDF membranes (Bio-Rad).

Primary antibodies used for detection are listed in Table 4, including their sources and working dilutions. Detection was carried out using HRP-conjugated secondary antibodies (anti-rabbit or anti-mouse; SigmaAldrich, St. Louis, MO, USA), and the chemiluminescent signal was developed using the Clarity Max™ Western ECL Substrate (Bio-Rad). Signal acquisition was performed using a ChemiDoc MP Imaging System (Bio-Rad).

Antibody specificity had been previously validated in human colorectal, pancreatic, and breast cancer cell lines [16] before their application to the CRC tissue homogenates in this study. Densitometric quantification of immunoreactive bands was conducted using ImageJ 1.54 M software (NIH, Bethesda, MD, USA), and β-actin was used as an internal loading control.

### 4.3. Biochemical Investigations

Serum aliquots were thawed before the analyses assessing metal concentrations and associated proteins. All assays on serum were performed in duplicate. Serum iron, transferrin, and ferritin concentrations were quantified using the Abbott ALINITY ci-series automatic biochemical analyzer (Abbott, Abbott Park, IL, USA). Serum iron was measured by a colorimetric method without deproteinization, transferrin was quantified using an immunoturbidimetric method, and ferritin was measured by a chemiluminescent microparticle immunoassay (CMIA). The % transferrin saturation was calculated by dividing the amount of serum iron by the total iron-binding capacity (TIBC), using the formula TIBC = transferrin × 102 × 1.25; thus, % transferrin saturation = (serum iron/TIBC) × 100. Ceruloplasmin concentration was measured by immunoturbidimetry (Futura System Srl, Rome, Italy), automated on a Pentra 400 analyzer (Horiba ABX, Montpellier, France). Purchased pooled healthy sera (IMCOM [high] and IMCONLOW [low]; Futura Systems) were used for assay validation.

Regarding testosterone, estradiol, progesterone, luteinizing hormone (LH), follicle-stimulating hormone (FSH), and carcinoembryonic antigen (CEA), all measurements were performed using CMIA. Pooled reference sera (Multichem S Plus; Technopath Clinical Diagnostics, Tipperary, Ireland) were used for assay validation.

### 4.4. Statistical Analyses

All statistical analyses were conducted using R (version 4.3.1) and Python (version 3.10, Jupyter environment) and SPSS (version 29.0, IBM SPSS Statistics for Windows). Continuous variables were first assessed for normality using the Shapiro–Wilk test. Based on the data distribution, comparisons between groups were performed using non-parametric Mann–Whitney U tests. Due to the known physiological influence of sex on both iron and hormonal pathways, all analyses involving serum biomarkers were stratified by sex. Within these sex-specific groups, age adjustment was applied selectively where appropriate (e.g., in male subgroups showing an age imbalance); comparisons were adjusted for age using linear regression residuals.

Correlations between serum biomarkers and clinical or molecular variables were evaluated using Spearman’s rank correlation coefficient. Correlation matrices were visualized using heatmaps. Where age adjustment was needed (e.g., in male subgroups), linear regression models including age as a covariate were used to extract residuals, and Spearman correlations were subsequently applied to these residuals to evaluate age-adjusted associations in a non-parametric framework. A *p*-value < 0.05 was considered statistically significant. All statistical testing was two-tailed.

## 5. Conclusions

Our findings reveal that CRC is associated with a systemic dysregulation of sex steroid hormones and iron metabolism, which correlates with the tumor stage and EMT-related features. These associations are sex-specific and suggest that circulating biomarkers may reflect biologically relevant mechanisms involved in tumor progression. Future studies are needed to validate these results and to explore their potential diagnostic and prognostic utility in personalized CRC management.

## Figures and Tables

**Table 1:** Group characteristics—CRC vs. controls.

	CRC (n = 82)	Control (n = 31)	Age and BMI Comparisons (*t*-Test)
**Age—mean ± SD**	69.34 ± 10.8	61.4 ± 7.2	*p* < 0.001
**Female (%)**	22%	34.0%	
**Male (%)**	78.0%	66.0%	*p* = 0.1380 *
**BMI**	25.49	24.28	*p* = 0.161
**Hemoglobin**	11.57 ± 1.97	14.5 ± 0.86	*p* < 0.0001

* Sex comparison (chi^2^ test).

**Table 2:** Comparison of serum sex hormone levels in CRC patients vs. controls, stratified by sex.

A. Sex Hormones and CEA Biomarkers					
Sex	Variable	Control Median (Range)	CRC Median (Range)	*p*-Value	Normal Reference Range
**Female**	**FSH (mIU/mL)**	68.55 [35.44–97.36]	51.80 [3.79–107.99]	0.0513	19.3–100.6
	**LH (mIU/mL)**	20.01 [7.81–34.92]	16.06 [0.00–39.61]	0.0556	19.3–100.6
	**Estradiol (pg/mL)**	12.00 [9.90–21.90]	16.95 [4.90–200.90]	0.005	0–39.5 pg/mL
	**Progesterone**	0.10 [0.09–0.20]	0.17 [0.09–16.10]	0.0077	0.15–0.8
	**Testosterone ng/dL**	0.92 [0.52–1.42]	0.89 [0.04–3.39]	0.915	0.3–1.9
	**CEA (ng/mL)**	2.12 [1.72–6.18]	1.94 [1.58–36.70]	0.4293	0–2.9
**Male**	**FSH (mIU/mL)**	4.70 [1.58–17.88]	7.45 [1.19–93.57]	0.5524	1.42–15.4
	**LH (mIU/mL)**	3.48 [0.20–6.90]	4.40 [1.36–35.00]	0.7021	1.42–15.4
	**Estradiol (pg/mL)**	27.53 [3.00–72.90]	26.00 [6.90–60.00]	0.5424	13.5–59.5 pg/mL
	**Progesterone (ng/mL)**	0.20 [0.10–4.91]	0.17 [0.09–2.17]	0.04	0.1–0.6
	**Testosterone**	17.69 [1.60–95.00]	14.32 [0.40–29.73]	0.5304	5–21 ng/dL
	**CEA (ng/mL)**	2.04 [1.54–4.64]	3.25 [1.72–75.65]	0.1059	0–2.9
**B. Iron-Related Biomarkers**					
**Sex**	**Variable**	**Control Median [Range]**	**CRC Median [Range]**	***p*** **-Value**	**Normal Reference Range**
**Female**	**Iron (µg/dL)**	91.50 [56.00–146.00]	35.00 [8.00–119.00]	<0.001	37–164
	**Ferritin (ng/mL)**	55.96 [9.48–174.49]	29.56 [3.30–3814.40]	0.1251	24–307
	**Transferrin (mg/dL)**	268.00 [209.0–314.00]	216.45 [60.00–338.90]	<0.001	200–360
	**% Transferrin saturation**	34.89 [18.83–47.16]	12.32 [1.66–53.07]	0.0052	15–50%
	**Ceruloplasmin (mg/dL)**	26.95 [23.70–27.50]	31.76 [20.31–43.80]	0.0309	20–60
	**Ceruloplasmin/Transferrin**	0.99 [0.83–1.09]	1.29 [0.11–3.55]	0.0222	0.05–0.21 ^1^
**Male**	**Iron (µg/dL)**	101.00 [40.00–151.00]	44.00 [9.00–346.00]	0.003	37–164
	**Ferritin (ng/mL)**	55.10 [8.20–287.00]	64.70 [5.40–1389.28]	0.2209	24–307
	**Transferrin (mg/dL)**	255.90 [46.73–315.90]	226.90 [131.00–346.90]	0.0484	200–360
	**% Transferrin saturation**	28.54 [11.32–45.66]	17.09 [2.36–82.85]	0.0091	15–50%
	**Ceruloplasmin (mg/dL)**	24.65 [20.90–34.14]	28.71 [22.28–45.90]	0.0209	20–60
	**Ceruloplasmin/Transferrin**	1.03 [0.70–5.26]	1.30 [0.11–2.93]	0.0406	0.05–0.2

For the male participants, age was included as a covariate in all the statistical models; for the female participants, no age correction was applied, since no significant age difference was observed between CRC patients and the controls; ^1^ Reference range value reported in [15].

**Table 3:** Comparison of epithelial–mesenchymal transition (EMT) markers in tumor vs. healthy mucosa.

EMT Biomarker	Tumor Mean ± SD	Healthy Mucosa Mean ± SD	Tumor Median (Range)	Healthy Mucosa Median (Range)	Test (Wilcoxon) *p*-Value
**MEMO1**	1.08 ± 0.60	1.04 ± 0.61	1.00 (0.00–3.16)	0.92 (0.10–3.31)	0.7321
**Vinculin**	0.75 ± 0.48	1.46 ± 0.98	0.67 (0.00–2.56)	1.25 (0.06–7.52)	<0.001
**Fibronectin**	1.68 ± 2.25	1.18 ± 1.23	0.96 (0.03–10.47)	0.81 (0.04–6.27)	0.2569
**E-cadherin**	1.44 ± 1.63	0.81 ± 0.54	1.07 (0.04–9.35)	0.71 (0.00–2.39)	<0.001
**Vimentin**	0.78 ± 1.45	4.08 ± 5.72	0.28 (0.00–10.51)	1.76 (0.00–29.16)	<0.001

Wilcoxon test for comparison between tumor tissue and the corresponding healthy mucosa.

**Table 4:** Primary antibodies used for the Western blot analysis.

Primary Antibody	Host	Supplier	Dilution
MEMO1	Mouse	Santa Cruz Biotechnology Inc. (Dallas, TX 75220, USA) #sc-517412	1:1000
E-cadherin	Mouse	BD Transduction Laboratories (Franklin Lakes, NJ, USA) #610181	1:1000
Fibronectin	Rabbit	Merck Life Science S.r.l (Milano, Italia) #F3648	1:1000
Vimentin	Mouse	Santa Cruz Biotechnology Inc. (Dallas, TX 75220, USA) #sc-373717	1:1000
Vinculin	Mouse	Santa Cruz Biotechnology Inc. (Dallas, TX 75220, USA) #sc-73614	1:2000

## Data Availability

The data presented in this study are available on request from the corresponding author. The data are not publicly available since patient recruitment is still in progress.
